# Polycyclic aromatic compounds as anticancer agents: synthesis and biological evaluation of methoxy dibenzofluorene derivatives

**DOI:** 10.3389/fchem.2014.00055

**Published:** 2014-08-01

**Authors:** Frederick F. Becker, Bimal K. Banik

**Affiliations:** Department of Translational Molecular Pathology, M. D. Anderson Cancer Center, The University of TexasHouston, TX USA

**Keywords:** methoxy dibenzofluorenes, aromatic nitration, *in vitro* tests, antitumor activity

## Abstract

Synthesis of a new methoxy dibenzofluorene through alkylation, cyclodehydration and aromatization in a one-pot operation is achieved for the first time. Using this hydrocarbon, a few derivatives are prepared through aromatic nitration, catalytic hydrogenation, coupling reaction with a side chain and reduction. The benzylic position of this hydrocarbon with the side chain is oxidized and reduced. Some of these derivatives have demonstrated excellent antitumor activities *in vitro*. This study confirms antitumor activity depends on the structures of the molecules.

## Introduction

Polyaromatic compounds are prepared by numerous methods (Clar, 1964; Harvey, 1997). Some of the methods are widely used in the synthesis of compounds containing multiple ring containing structures all of which are not aromatic. Numerous polyaromatic compounds have demonstrated carcinogenic and mutagenic activities. The causes of these adverse activities of these compounds have been realized through different hypothetical mechanisms and theories (Di Raddo and Chan, 1982). Most of the past research on polyaromatic compounds is mainly based upon two important areas: synthesis and carcinogenicity/mutagenicity. A few suitably substituted polycyclic aromatic compounds have demonstrated anticancer activities, but their mechanism of action is not established. For example, the anticancer activity of these compounds may be due to their intercalating properties or covalent binding abilities to DNA (Palmer et al., 1988; Denny et al., 1990). In addition, cell membrane interaction of these compounds is also proposed as their mechanism of actions. Our study has indicated that substituted chrysenes act on the cancer cell through interactions with the cell membrane (Becker and Banik, 1998).

Since some polyaromatic compounds have demonstrated carcinogenic and mutagenic properties, the development of such compounds as antitumor drugs may raise concerns. However, many clinically active anticancer drugs that are not derived from polyaromatic compounds are carcinogenic and other harmful properties. Benzene is highly carcinogenic, but many compounds derived from benzene are life-saving drugs. It has been shown that alteration of the structure of polyaromatic compounds can help to interact with specific organelles to evoke selective cytotoxic reactions (Palmer et al., 1992; Cherubim et al., 1993). This simple, but very crucial concept is used by many scientists and as result of these approaches many carbazoles, anthracenes, and related structures are in current clinical use (Iyengar et al., 1997; Brana et al., 2001; Martinez and Chacon-Garcia, 2005; Landis-Piwowar et al., 2006; Rescifina et al., 2006; Bandyopadhyay et al., 2012). Despite huge progress in the identification of numerous active cancer chemotherapeutic agents, synthesis and biological evaluation of new methoxy dibenzofluorene derivatives has not been reported. The skeleton present in this molecule is highly suited for several structural alterations. The pentacyclic aromatic rings may also interact with cell membranes as we have observed in our study with chrysene derivatives. Moreover, the 13-position of this type of ring system is available for substitution. The active CH_2_ group present in the 13-position may create cation, anion and radical. Thus, it would be highly important to study the effects of functionalized methoxy dibenzofluorenes as new anticancer agents (Becker et al., 2000). We report herein our preliminary findings that uncover anticancer activities that depend on the groups present in these molecules.

## Results and discussions

**Scheme 1:**
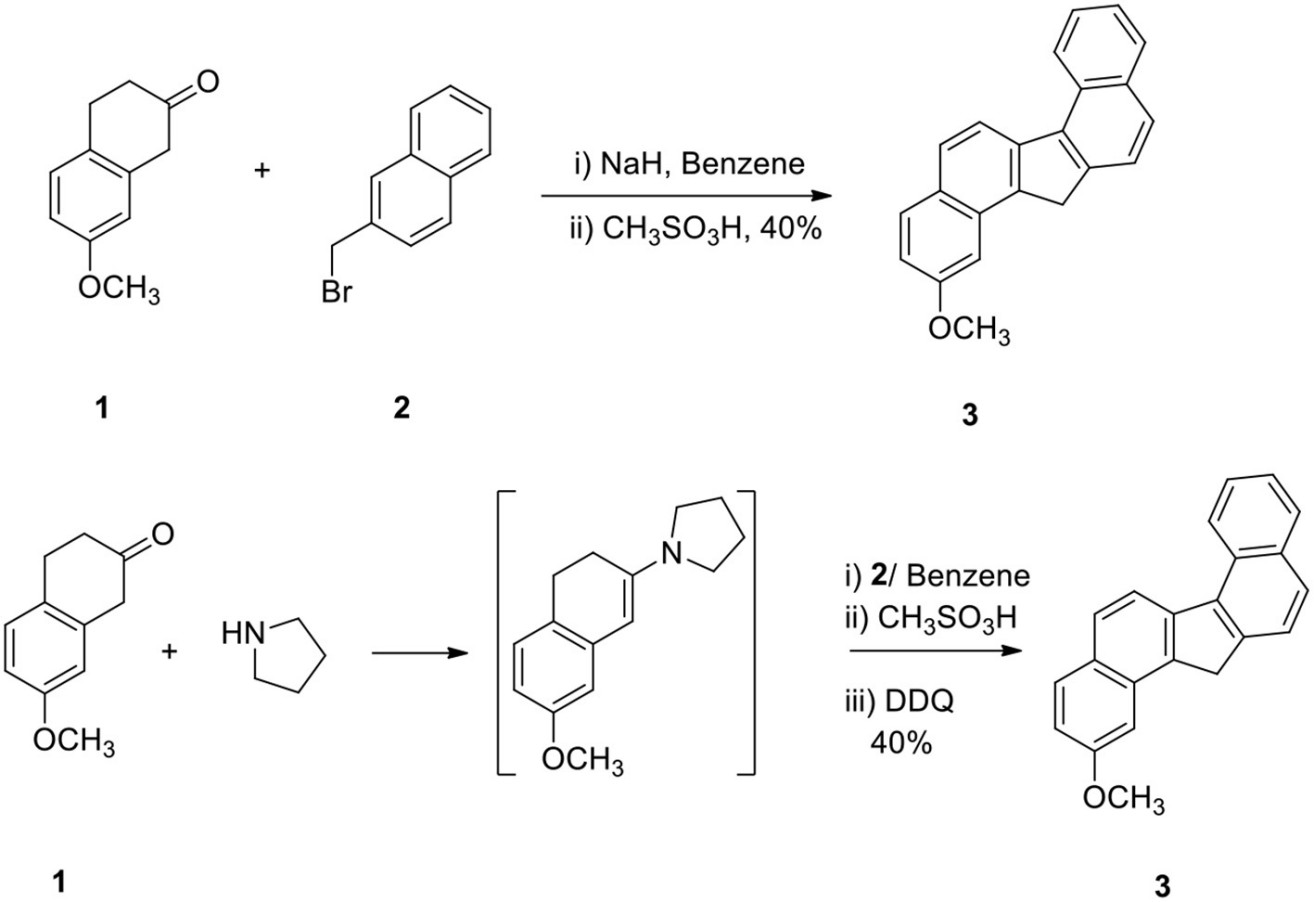
**Synthesis of methoxy dibenzofluorene**.

The reaction of 2-methoxy β-tetralone (**1**) with β-methyl naphthalenyl bromide (**2**) in the presence of sodium hydride in benzene at −10°C for 1 h and treatment of the resulting intermediate with methane sulfonic acid for additional 3 hours afforded 2-methoxy dibenzo[a,g]fluorene (**3**) in 40% yield in a one-pot operation (Scheme 1). This method is very simple since alkylation, cyclodehydration and aromatization take place simultaneously. This method demonstrates a powerful strategy for the preparation of methoxy dibenzo[a,g]fluorene.

In another method, compound **1** was converted to enamine, alkylated the enamine with **2** and the intermediate was then cyclized and aromatized to **3** (Scheme 1). The yield of the product **3** obtained by these two methods was comparable.

**Scheme 2:**
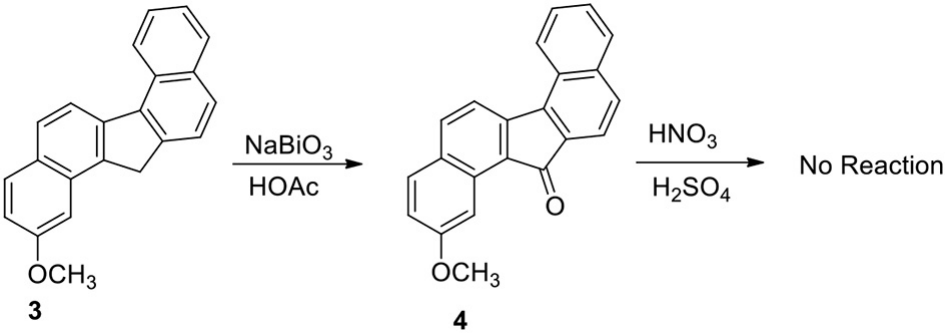
**Oxidation and nitration study of methoxy dibenzofluorene**.

Our plan was to add a linker with 4-carbon chain that have a basic nitrogen unit at the terminal site to any carbon at **3** and oxidize the benzylic methylene group. However, the plan was not straight forward. Oxidation of **3** to benzylic ketone **4** by sodium bismuthate was achieved (Scheme 2). However, ketone **4** failed to produce nitro compounds under different reaction conditions with nitric acid and metal salts (Samajdar et al., 2000; Bose et al., 2007). The keto group deactivated the aromatic ring systems significantly and as a results no nitration of the aromatic ring was possible.

Functionalization of monocyclic and bicyclic aromatic compound through electrophilic nitration is explored by many methods. The failure of compound **4** to undergo nitration appears to be the result of deactivation exerted by the keto group present in the bridged system of the ring. On the basis of the deactivation hypothesis, we performed nitration of the hydrocarbon **3** with nitric acid/sulfuric acid mixture and by bismuth nitrate-induced clay impregnated reactions.

**Scheme 3:**
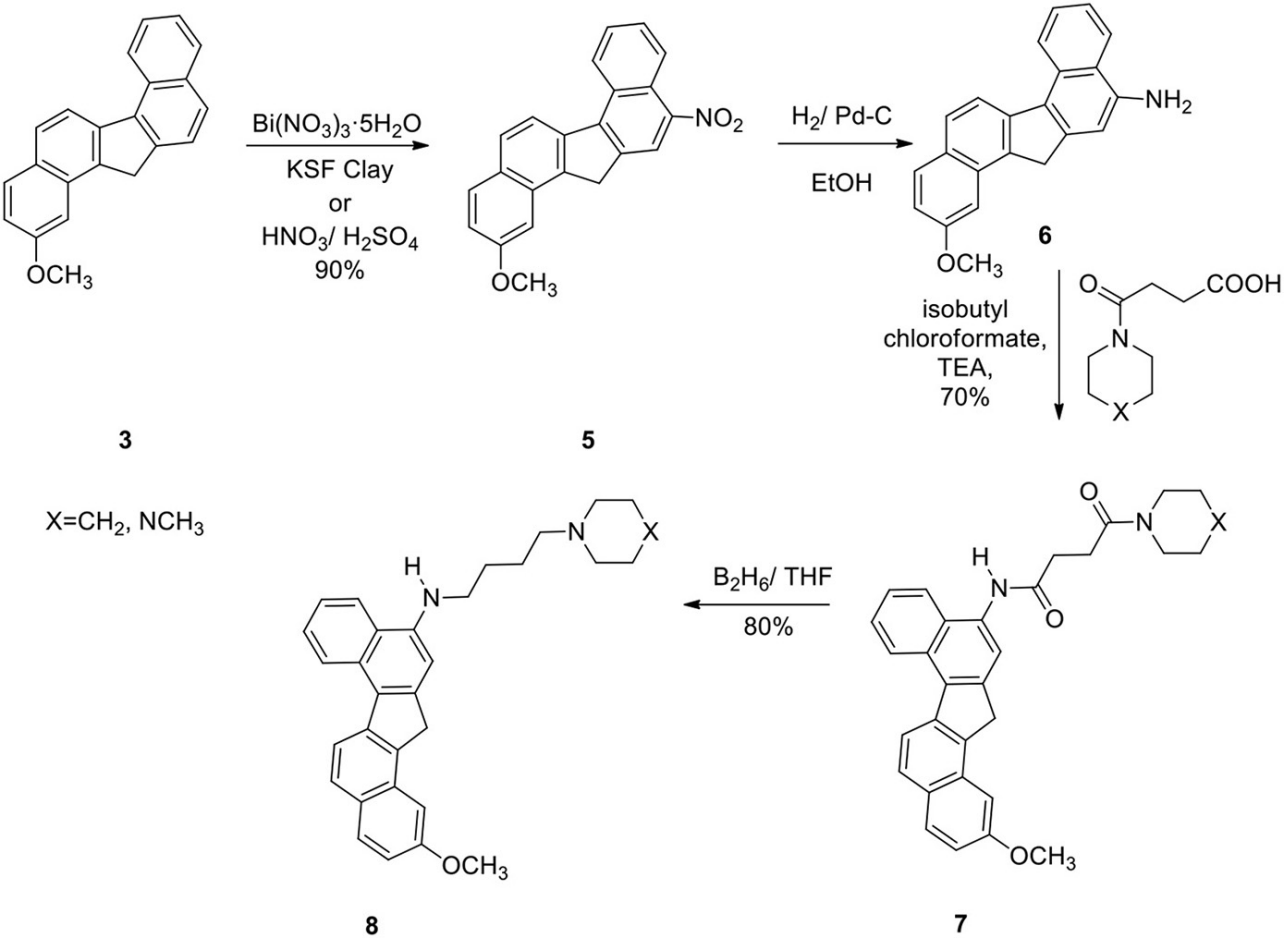
**Synthesis of methoxy dibenzofluorene derivatives with amine side chain**.

**Scheme 4:**
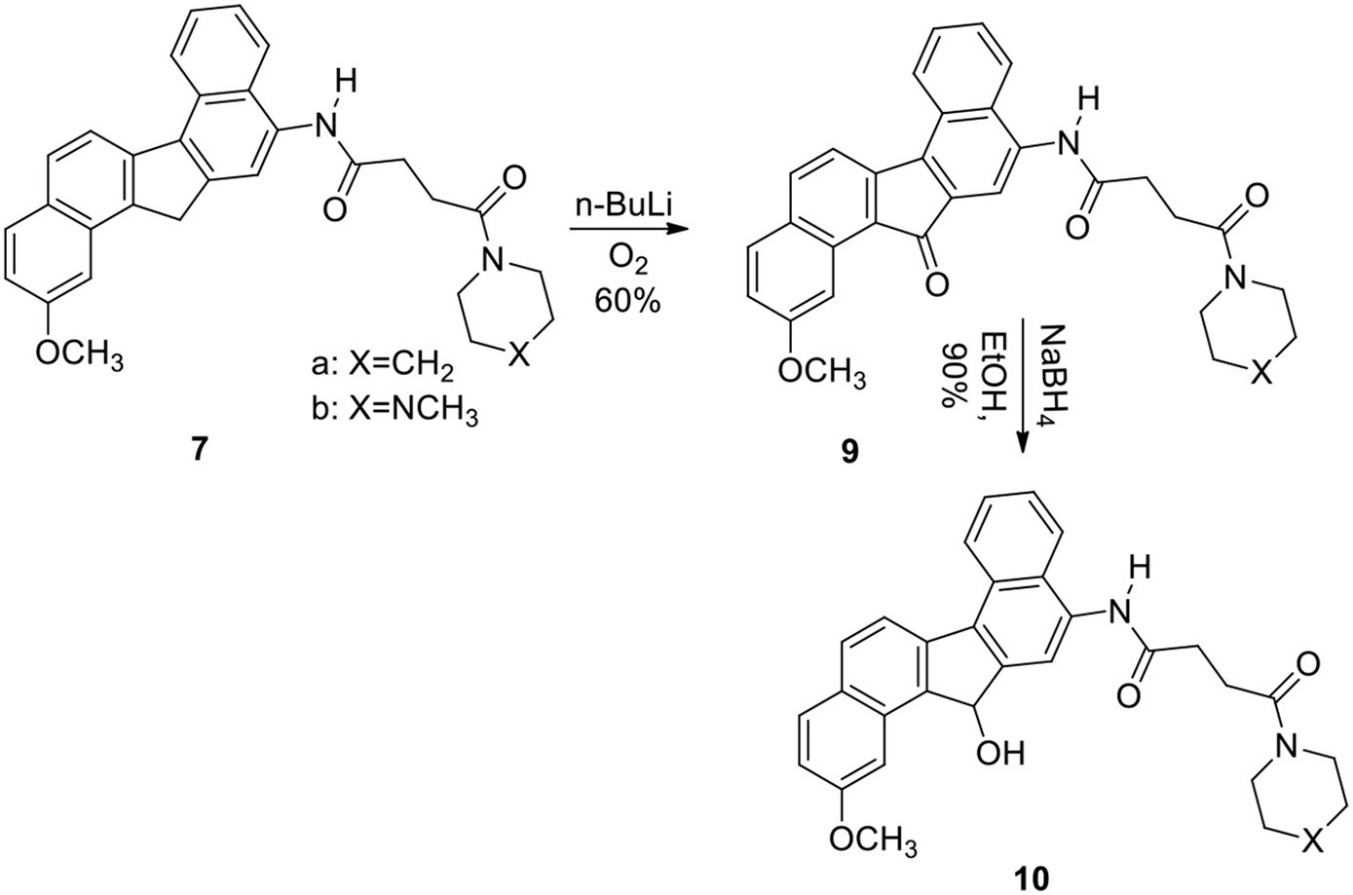
**Synthesis of methoxy dibenzofluorene derivatives with amide side chain**.

The reaction was successful and the product was a single nitro derivative **5** obtained in excellent yield. The position of the nitro group in the aromatic ring was assigned by NMR spectra and comparison with our previous compounds that have no methoxy groups. It was important to note that nitration takes place at the unsubstituted aromatic ring. Several methods were attempted to reduce the nitro group in **5**. Thus reduction of **5** with hydrogen gas/Pd-C, hydrazine/Pd-C, samarium/iodine, and indium/ammonium chloride were effective in producing the corresponding amino compound **6**. The amine **6** was then reacted with the acids described earlier in the presence of isobutylchloroformate and triethylamine (Becker and Banik, 1998). The diamides **7a** (X=CH_2_) and **7b** (X=NCH_3_) were obtained in 70% yield. On oxidation, the methylene group in **7** produced the ketone **9**. The ketone **9** was reduced to the alcohol **10**. Diborane was used to reduce the amide groups in **7** to **8** (Schemes 3 and 4).

These compounds were tested in the University of Texas M. D. Anderson Cancer Center's Core Analytical Laboratory against eight tumor lines all of which have been used in the NCI panel for the testing of chemotherapeutic agents. The antitumor activity of these new methoxy diben-zofluorene derivatives **7** to **10** were performed and compared with cisplatin (Table 1). The results of these tests are interesting, and indicate the significance of altering structures in terms of antitumor activities. Compounds **7b, 8a** and **8b** were more active than cisplatin in many of these cancer cell lines.

**Table 1:** **IC_50_ (micro molar) of compounds 7 to 10 MTT assay (72 h continuous exposure)^a^**.

**Cell lines**	**Cisplatin**	**7a**	**7b**	**9a**	**9b**	**10a**	**10b**	**8a**	**8b**
B-16	7.33	128.50	4.30	>100	12.30	28.70	11.08	4.05	1.67
BRO	5.66	103.54	3.89	>100	12.67	31.74	12.41	4.26	3.54
HL-60	-	33.33	3.48	74.18	7.33	12.00	4.19	-	-
L-1210	-	53.70	3.98	78.36	25.85	16.44	7.05	-	-
MCF-7	15.99	50.63	4.32	78.30	13.70	20.11	11.85	4.82	4.56
OVCAR-3	-	94.32	4.11	98.00	2.61	19.77	8.66	3.12	1.80
PC-3	1.66	32.52	3.78	31.45	27.32	13.88	6.56	4.49	4.39
HT-29	15.99	>100	3.86	61.31	21.66	9.98	13.67	4.11	3.27

a *All data were provided as IC_50_ values (micro molar) and assays were conducted by 72 h continuous exposure by the MTT method. The final concentration of solvent was <0.625% which was not toxic to the cells. All dilutions were made in RPMI 1640 with 10% FBS. The cytotoxity data is based on at least three separate experiments with deviations within 0.2 μmol*.

In fact, **7b, 8a**, and **8b** are some of the most active molecules that we have derived from the study of polyaromatic compounds. The keto group at position 13 created the fluorenone **9** with reduced activity. It was clear that the piperidine group at the terminus of the alkyl chain decreases activity compared with those that terminate with the N-methyl piperazine. Clearly, this was observed in compounds **9** and **10**. The alcohol group at position 13 in compound **10b** produced a reduced activity when compared with **7b**. Reduction of **7** resulted in their amine derivative **8**. An impressive increase was seen in the amine derivatives **8a** and **8b** regardless of the terminus units. The cause of the spectacular differences of the activity of the two series of compounds in all cell lines is not known. A similar observation was noted with demethoxy analogs of the dibenzofluorene and chrysene derivatives. Amino compounds were more potent than amides in these two series of molecules. This was confirmed by the fact that compounds **8a**, **8b** and **7b** are the more cytotoxic compared to **7a**. A slight increase in activity in **10b** compared to **10a** and **9b** compared to **9a** was also observed. The introduction of at one basic group (tertiary amine) at the terminal site of these molecules was probably responsible for the increased activity. The basic units will help to increase the pKa value so that these molecules can be protonated at physiological pH. These type of polyaromatic highly lipophilic and cationic molecules are expected to bind to several anionic structures inside cells such as to phosphate groups of nucleic acids (DNA and RNA). These lipophilic molecules are also expected to interact strongly with the lipids components in cell membrane. The *in vitro* cytotoxicity of these new molecules are similar or better than that of cisplatin which act through a different mechanism of action primarily through cell damage and death. However, it is also known that activity *in vitro* is not proportional to clinical applications in many instances. For example, adriamycin is one of the most potent anticancer drugs *in vitro* and *in vivo*, but it is also established that it is not a successful clinically active agent.

Notably, the aromatic methoxy group present in the molecules as described herein can be used for a detail structure-activity relationships study. For example, the aromatic methoxy group can be converted to a phenolic hydroxyl group and phenolic hydroxyl group can be alkylated or acylated with different types of side chains. In addition the phenolic hydroxyl group can be further used to perform a number of electrophilic substitution (halogenation, nitration and sulfonation).

## Experimental (general methods)

All reactions described in this paper were carried out under a well-ventilated hood. Dichloromethane and tetrahydrofuran were dried and freshly distilled before use. IR spectra were recorded on a Perkin Elmer instrument and UV spectra were recorded on a Perkin Elmer instrument. NMR spectra were recorded on Bruker 200 and 300 MHz spectrometers. Chemical shifts were recorded as delta values in parts per million downfield from tetramethyl silane as the internal standard in CDCl_3_. Mass spectra were obtained on a Micromass VG platform with a single quadrapole and fitted with an electrospray source. Elemental analyses were performed by Schwarzkopf Microanalytical Laboratory, Inc., New York. Melting points were taken in open capillary tube. Column chromatography was carried out with Aldrich silica gel (230 mesh). TLC was run with pre-coated silica gel plate. Sodium sulfate was used as the drying agent after all extractions.

### 2-methoxy-13^/^H-dibenzofluorene (3)

Sodium hydride (10 g) in dry benzene (30 mL) was taken in a flask and it was cooled at 0° to −10°C. 2-Methoxy β-tetralone **1** (5 g) dissolved in dry benzene (25 mL) was added to it in an inert atmosphere and the reaction mixture was stirred for 30 min. β-Methyl naphthalenyl bromide (**2**, 1.5 equivalent with respect to ketone) in benzene was added to the enolate at cold conditions and the reaction mixture was stirred at 0°C for additional 1 h. Methane sulfonic acid (20 mL) was added carefully to the reaction mixture and it was then refluxed for 3 h. Water (50 mL) was added to the reaction mixture and benzene layer was collected. The organic part was washed with aqueous NaOH solution (2%, 25 mL) and dried. On evaporation of the solvent, hydrocarbon **3** was obtained in 40% yield. This was used directly for the next step without purification.

### 2-methoxy-11-nitro-13^/^H-dibenzofluorene (5)

To an ice-cold solution of the hydrocarbon **3** (500 mg) in THF (20 mL) was added glacial acetic acid (25 mL). Nitric acid (90%, 10 mL) was added drop wise to the solution at ice-cold temperature. After the addition was complete, stirring was continued for additional 2 h at room temperature. The reaction mixture was then poured into crushed ice, filtered, and washed with water until free from acid. The crude product **5** (80%) was used directly for the next step.

### 2-methoxy-11-amino-13^/^H-dibenzofluorene (6)

Nitro compound **5** (400 mg) was hydrogenated with 10% Pd/C (50 mg) in ethanol for 24 h to afford the amine **6** (90%); IR (CH_2_Cl_2_): 3430, 3355, 3050, 2955, 2920, 2838, 1695, 1618, 1585, 1510, 1440 cm^−1^; ^1^H NMR (200 MHz): δ 8.80–8.76 (1H, d), 8.40–8.34 (1H, d), 8.10–7.80 (3H, m), 7.69–7.43 (3H, m), 7.15 (1H, s), 7.02 (1H, s), 4.18 (2H, s), 3.72 (s, 3H); ^13^C NMR (400 MHz): δ 142.5, 142.0, 140.7, 140.0, 139.0, 131.2, 130.2, 128.7, 127.3, 126.4, 126.1, 125.2, 124.3, 124.1, 124.0, 123.5, 123.3, 121.7, 120.8, 107.7, 37.4; 30.05; Mass: 312 (M+H)^+^.

### 2-methoxy-N-[2^/^-(13^/^H-dibenzo[a,g]-fluorenyl)]-4-(1^/^-piperidinyl)-butane-1,4-dicarboxiamide (7a)

To an ice-cold solution of the acid described in Scheme 3 (1.0 g) in dry CH_2_Cl_2_ (50 mL) was added dry triethylamine (0.8 mL) followed by freshly distilled isobutyl chloroformate (0.6 mL). Stirring was continued in cold conditions for 10 min. Next this mixed anhydride was added drop wise to an ice-cold solution of the amine **6** (700 mg) in dry CH_2_Cl_2_ (50 mL) and stirring was continued overnight. The reaction mixture was washed successively with HCl (5%), NaHCO_3_(5%), brine and then dried. Removal of the solvent under reduced pressure afforded the crude product, which was purified by column chromatography over silica gel to obtain **7a** (70%); mp 180–181°C; IR (CH_2_Cl_2_): 3260, 2956, 1650, 1540, 1500, 1452, 1279, 80, 735 cm^−1^; ^1^HNMR (200 MHz): δ 9.42 (1H, s, ArNHCO-), 8.86 (*J* = 8.42 Hz, 1H, d, Ar), 8.47 (*J* = 8.68 Hz, 1H, d, Ar), 8.39 (1H, s, Ar), 8.15 (*J* = 8.35 Hz, 1H, d, Ar), 8.05 (*J* = 8.03 Hz, 1H, d, Ar), 7.95 (*J* = 9.29 Hz, 2H, d, Ar), 7.60–7.40 (2H, m, Ar), 7. 35 (1H, s, Ar), 4.30 (2H, s, benzylic CH_2_), 3.81 (3H, s, OCH_3_), 3.75–3.60 (2H, brt, -CONCH_2_), 3.55–3.36 (2H, brt,-CONCH_2_), 3.04–2.74 (4H, m, -COCH_2_CH_2_CO-), 1.80–1.42 (6H, brs, -NCH_2_(CH_2_)_3_CH_2_-); ^13^C NMR: δ 171.9, 169.2, 140.2, 138.9, 138.6, 137.9, 133.5, 130.8, 129.9, 129. 6, 128.5, 126.8, 126. 2, 125.4, 125.0, 124.8, 124.3, 124.0, 123.2, 121.4, 121.0, 116.4, 45.5, 43.4, 42.1, 36.0, 32.4, 28.9, 25.4, 24.8, 23.3; Mass: 479 (M+H)^+^; Anal. calcd for C_31_H_30_N_2_O_3_: C, 77.82; H, 6.27; N, 5.85%. Found: C, 77.70; H, 6.02; N, 6.02%.

### 2-methoxy-N-[2^/^-(13^/^H-dibenzo[a,g]fluorenyl)]-4-(4^/^-N-methyl-piperazinyl)-butane-1,4-dicarboxiamide (7b)

70%; mp 208–210°C; IR (neat): 3260, 2925, 1642, 1538, 1441, 1290, 1258, 1145, 809, 745 cm^−1^; ^1^H NMR (200 MHz): δ 9.12 (1H, s, ArNHCO-), 8.80 (*J* = 8.40 Hz, 1H, d, H7), 8.45 (*J* = 8.70 Hz, 1H, d, Ar), 8. 42 (1H, Ar), 8.29 (1H, s, Ar), 8.10 (*J* = 8.32 Hz, 1H, d, Ar), 8.05 (*J* = 8.03 Hz, 1H, d, Ar), 7.94 (*J* = 8.00 Hz, 2H, d, Ar), 7.71–7.37 (3H, m, Ar), 4.22 (2H, s, benzylic CH_2_), 3.90 (3H, s), 3.81–3.64 (2H, brt, -CONCH_2_), 3.60–3.46 (2H, brt, -CON-CH_2_), 3.00–2.74 (4H, m,-COCH_2_CH_2_CO-), 2.54–2.20 (6H, m, -(CH_2_)_2_NCH_3_, with a singlet at δ 2.32 for - NCH_3_); ^13^C NMR (300 MHz): δ 171.1, 169.5, 142.0, 140.8, 139.5, 134.2, 132.7, 131.6, 129.4, 128.8, 128.4, 127.3, 126.2, 125.9, 125.1, 124.8, 124.7, 124.6, 124.0, 122.2, 121.4, 117.6, 54.7, 54.3, 46.0, 45.3, 42.4, 42.1, 36.1, 32.9, 29.3; Mass (M+H)^+^: 494; Anal. calcd for C_31_H_31_N_3_O_3_: C, 75.45; H, 6.28; N, 8.51%. Found: C, 75.48; H, 6.05; N, 8.43%.

### 2-methoxy-N-[2^/^-(13^/^H-dibenzo[a,g]-fluorene-13^/^-one]-4-(1^/^-piperidinyl)-butane-1,4-dicarboxiamide (9a)

To a solution of **7a** (650 mg) in dry THF (40 mL) at −78°C under argon was added a solution of n-BuLi (1.6 mL, 2.5 M) in cyclohexane. The deep-yellow colored solution was stirred for 2 h at this temperature. Dry O_2_ was bubbled through the solution for 1 h. The temperature was allowed to rise to room temperature while O_2_ continued to bubble through the solution for an additional 3 h. The reaction was quenched with water (20 mL) and CH_2_Cl_2_ (20 mL), and stirring was continued for 30 min. The organic layer was collected, washed with brine, and dried. On evaporation of the solvent, the crude product was obtained, which was crystallized from CH_2_Cl_2_/hexanes to yield **9a** (60%); mp 220–222°C; IR (neat): 3240, 2936, 1700, 1645, 1520, 1450, 1264, 810, 802, 732, 702 cm^−1^; ^1^H NMR (200 MHz): δ 9.45 (1H, s, ArNHCO-), 8.91 (*J* = 8.40 Hz, 1H, d, Ar), 8.48–8.45 (1H, m, Ar), 8.20–8.05 (2H, m, Ar), 7.88 (*J* = 8.45 Hz, 1H, d, Ar), 7.72 (*J* = 8.18 Hz, 1H, d, Ar), 7.56–7.44 (2H, m, Ar), 7.35 (*J* = 8.06 Hz, 1H, d, Ar), 3.80–3.62 (2H, brt, -CONCH_2_), 3. 78 (3H, s, OCH_3_), 3.60–3.37 (2H, brt, -CONCH_2_), 2.85 (4H, s,-COCH_2_CH_2_CO-), 1.77–1.48 (6H, brs, -NCH_2_(CH_2_)_3_CH_2_-); ^13^C NMR (300 MHz, CDCl_3_): δ 194.8, 170.8, 170.2, 147.0, 140.2. 138.1, 135.2, 133.9, 132.5, 131.0, 130.8, 129.2, 128.3, 128.0, 127.2, 127.1, 126. 4, 126.1, 125.2, 124.8, 123.8, 123.2, 119.9, 115.0, 46.5, 44.7, 26.5, 26.2, 24.2, 22.1, 14.3; Mass (M+H)^+^: 493; Anal. calcd for C_31_H_28_N_2_O_4_: C, 75.60; H, 5.69; N, 5.69%. Found: C, 75.51; H, 5.62; N, 5.54%.

### 2-methoxy-N-[2^/^-(13^/^H-dibenzo[a,g]-fluorene-13^/^-one]-4-(4^/^N-methyl-piperazinyl)-butane-1,4-dicarboxiamide (9b)

60%; mp 210–211°C; IR (neat): 3235, 2925, 2850, 1700, 1648, 1545, 1520, 1460, 1268, 1258, 1000, 810, 752, 704 cm^−1^; ^1^H NMR (200 MHz): δ 9.15 (1H, s, ArNHCO-), 8.94 (*J* = 8.57 Hz, 1H, d, Ar), 8.48–8.35 (1H, m, Ar), 8.33–7.87 (2H, m, Ar), 7.82 (*J* = 8.54 Hz, 1H, d, Ar), 7.70 (*J* = 8.33 Hz, 1H, d, Ar), 7.58–7.40 (3H, m, Ar), 7.40 (*J* = 7.82 Hz, 1H, d, Ar), 3.82–3.38 (4H, m, CON(CH_2_)_2_, 3.77 (s, 3H), 2.95 (4H, s, -COCH_2_CH_2_CO-), 2.61–2.19 (7H, m, (-CH_2_)_2_NCH_3_ with a singlet at δ 2.34 for-NCH_3_); ^13^C NMR (300 MHz): δ 196.0, 171.2, 171.0, 146.8, 138.2, 135.2, 135.8, 134.1, 133.8, 132.5, 130.7, 129.6, 128.4, 128.2, 128.0, 127.5, 126.6, 126.2, 124.5, 124.1, 123.4, 120.9, 114.4, 54.9, 54.2, 48.9, 45.9, 45.3, 32.6, 23.1, 14.3; Mass (M+H)^+^: 508; Anal. calcd for C_31_H_29_N_3_O_4_: C, 73.37; H, 5.71; N, 8.28%. Found: C, 73.20; H, 5.87; N, 8.12%.

### 2-methoxy-N-[2^/^-(13^/^H-dibenzo[a,g]-fluorene-13^/^-hydroxy]-4-(10-piperidinyl)-butane-1,4-dicarboxiamide (10a)

To a solution of **9a** (200 mg) in absolute EtOH (20 mL) was added NaBH_4_ (50 mg) at ice-cold temperature and the solution was stirred under this condition for 30 min. The temperature was allowed to rise to room temperature, and stirring was continued for an additional 4 h. The reaction was quenched by addition of water (10 mL) and the reactants were extracted with CH_2_Cl_2_ (50 mL), washed with brine and dried. On removal of the solvent, crude product was crystallized from CH_2_Cl_2_/hexanes to yield 140 mg (90%) of pure **10a**; mp 225–228; IR (neat): 3440, 3260, 2940, 2860, 1650, 1625, 1540, 1446, 1270, 1260, 1170, 1090, 810, 750 cm^−1^; ^1^H NMR (200 MHz): δ 9.25 (1H, s, ArNHCO-), 8.70 (*J* = 8.62 Hz, 1H, d, Ar), 8.39 (*J* = 8.07 Hz, 1H, d, Ar), 8.18–8.12 (2H, m, Ar), 8.05–7.85 (2H, m, Ar), 7.58–7.22 (4H, m, Ar), 5.85 (*J* = 10.28 Hz, 1H, d), 3.80 (3H, s, OCH_3_), 3.78–3.52 (2H, brt, -CONCH_2_), 3.50–3.38 (2H, brt,-CONCH_2_), 3.05–2.66 [5H, m, -COCH_2_CH_2_CO- with a doublet at δ 2.75 (*J* = 10.28 Hz which disappeared with D_2_O)], 1.75–1.42 [6H, brs, -NCH_2_(CH_2_)_3_CH_2_-]; Mass (M+H)^+^: 495; Anal. calcd for C_31_H_30_N_2_O_4_: C, 75.30; H, 6.07; N, 5.66%. Found: C, 75.34; H, 5.97; N, 5.56%.

### 2-methoxy-N-[2^/^-(13^/^H-dibenzo[a,g]-fluorene-13^/^-hydroxy]-4-(4^/^N-methyl-piperazinyl)-butane-1,4-dicarboxiamide (10b)

90%; mp 175–177°C; IR (neat): 3450, 3265, 2800, 1630, 1545, 1502, 1442, 1300, 1295, 1252, 1190, 1132, 1005, 818, 755 cm^−1^; ^1^H NMR (200 MHz): δ 9.10 (1H, s, ArNHCO-), 8.56 (*J* = 8.48 Hz, 1H, d, Ar), 8.39 (*J* = 8.26 Hz, 1H, d, Ar), 8.29 (*J* = 8.14 Hz, 1H, d, Ar), 8.15 (1H, s), 8.11–7.76 (2H, m, Ar), 7.60–7.4 (4H, m, Ar), 5.81 (1H, s), 3.80 (3H, s), 3.75–3.22 (4H, m, -CON(CH_2_)_2_-), 2.82-2.55 (4H, m,-COCH_2_CH_2_CO-), 2.73 (d, IH, disappeared with D_2_O, 2.38-2.12(7H, m, (-CH_2_)_2_NCH_3_, with a singlet at δ 2.11 for -NCH_3_); Mass (M+H)^+^: 510; Anal. calcd for C_31_H_31_N_3_O_4_: C, 73.08; H, 6.09; N, 8.25%. Found: C, 73.10; H, 6.11; N, 8.08%.

### 2-methoxy-N-[2^/^-(13^/^H-dibenzo[a,g]-fluorenyl]-4-(10-piperidinyl)-butane-1,4-diamine (8a)

To a solution of **7a** (50 mg) in THF (50 mL) were added borane-methyl sulfide complex (70 mL, 5 M) solution in diethyl ether under argon at ice-cold temperature, and the mixture was refluxed for 16 h. Then, HCl (15 mL, 5%) was added, and the mixture was refluxed for another 10 h. The solution was cooled, added to 1 M sodium hydroxide solution, extracted with ethyl acetate, washed with brine, and dried. On removal of the solvent, the crude diamine solidified, which was crystallized from ethyl acetate: hexanes (20:80) to give the pure diamine **8a** (80%); mp 170–171°C; IR (neat): 3385, 2939, 2755, 1630, 1620, 1581, 1560, 1545, 1468, 1453, 1348, 1278, 1178, 1110, 1050, 800, 770 cm^−1^; ^1^H NMR (200 MHz): δ 8.85 (*J* = 8.11 Hz, 1H, d), 8.39 (*J* = 8.70 Hz, 1H, d), 8.14–7.90 (3H, m, Ar), 7.66–7.30 (4H, m, Ar), 6.95 (1H, s), 4.28 (2H, s), 3.82 (3H, s), 3.48 (*J* = 6.60 Hz, 2H, t, ArNHCH_2_-), 2.66–2.35 [6H, brt, H_2_C-N(CH_2_)_2_-], 1.96–1.40 (10H, m); ^13^C NMR (300 MHz): δ 145.0, 143.3, 141.4, 138.8, 137.5, 130.6, 130.1, 129.1, 128.8, 128.1, 126.2, 125.9, 125.2, 124.6, 124.2, 124.0, 123.9, 122.1, 121.7, 120.9, 101.4, 58.2, 54.5, 50.2, 48.3, 44.1, 37.4, 27.1, 26.1, 24.4, 24.2; Mass (M+H)^+^: 451; Anal. calcd for C_31_H_34_N_2_O: C, 82.66; H, 7.55; N, 6.22%. Found: C, 82.50; H, 7.41; N, 6.13%.

### 2-methoxy-N-[2^/^-(13^/^H-dibenzo[a,g]-fluorenyl)]-4-(4^/^N-methylpiperazinyl)-butane-1,4-diamine (8b)

80%; mp 155–157°C; IR (neat): 3400, 2930, 2850, 1622, 1590, 1565, 1508, 1410, 1348, 1246, 1155, 1018, 810, 745 cm^−1^; ^1^H NMR (200 MHz): δ 8.85 (*J* = 8.35 Hz, 1H, d), 8.39 (*J* = 8.70 Hz, 1H, d), 8.10–7.86 (4H, m, Ar), 7.76–7.28 (4H, m, Ar), 6.98 (1H, s), 4.28 (2H, s), 3.78 (3H, s), 3.40 (*J* = 6.60 Hz, 2H, t, ArNHCH_2_-), 2.71–2.35 [10H, brt, (5-NCH_2_)], 2.32 (3H, s), 1.99–1.58 (4H, m); ^13^CNMR (300 MHz): δ 144.1, 142.9, 141.4, 139.1, 131.2, 131.0, 130.1, 129.8, 128.4, 127.0, 126.6, 126.2, 124.8, 123.9, 123.3, 122.8, 122.3, 121.1, 120.9, 101.4, 55.1, 54.7, 53.2, 48.8, 46.1, 44.4, 40.2, 36.9, 29.9, 28.1, 24.8132; Mass (M+H)^+^: 466; Anal. calcd for C_31_H_35_N_3_O: C, 80.17; H, 7.32; N, 9.05%. Found: C, 80.04; H, 7.45; N, 9.14%.

## Conclusion

Despite significant progress in the identification of novel cancer chemotherapeutic agents, synthesis and biological evaluation of new methoxy dibenzofuorene derivatives is not known. The synthesis of this aromatic hydrocarbon through one-pot method may receive well attention. We have demonstrated an electrophilic nitration of 2-methoxy-13H-dibenzo[a,g]-fluorene for the first time. Despite a complex structure and number of available sites, regioselective nitration of methoxy dibenzofluorene is interesting. The crucial antitumor activity of the several new compounds against a number of cancer cell lines is promising and this will open up a possibility of conducting a more detail structure-activity relationship study of other analogues that will be derived through chemical manipulation.

### Conflict of interest statement

The authors declare that the research was conducted in the absence of any commercial or financial relationships that could be construed as a potential conflict of interest.
